# Effects of Moderate Chronic Food Restriction on the Development of Postprandial Dyslipidemia with Ageing

**DOI:** 10.3390/nu11081865

**Published:** 2019-08-10

**Authors:** Alejandro Fernández, Lorena Mazuecos, Cristina Pintado, Blanca Rubio, Virginia López, Alain J. de Solís, María Rodríguez, Antonio Andrés, Nilda Gallardo

**Affiliations:** 1 Regional Centre for Biomedical Research (CRIB), University of Castilla-La Mancha, 13071 Ciudad Real, Spain; 2 Biochemistry Section, Faculty of Science and Chemical Technologies, University of Castilla-La Mancha, Avda Camilo José Cela 10, 13071 Ciudad Real, Spain; 3 Biochemistry Section, Faculty of Enviromental Science and Biochemistry, University of Castilla-La Mancha, Avda Carlos III s/n, 45071 Toledo, Spain; 4 Max Planck Institute for Metabolism Research, Gleueler Strasse 50, 50931 Cologne, Germany

**Keywords:** oral lipid loading test, ageing, postprandial hypertrigliceridemia, ChREBP, postprandial thermogenesis, adipose tissue

## Abstract

Ageing is a major risk factor for the development of metabolic disorders linked to dyslipidemia, usually accompanied by increased adiposity. The goal of this work was to investigate whether avoiding an excessive increase in adiposity with ageing, via moderate chronic food restriction (FR), ameliorates postprandial dyslipidemia in a rat model of metabolic syndrome associated with ageing. Accordingly, we performed an oral lipid loading test (OLLT) in mature middle-aged (7 months) and middle-old-aged (24 months) Wistar rats fed ad libitum (AL) or under moderate FR for 3 months. Briefly, overnight fasted rats were orally administered a bolus of extra-virgin olive oil (1 mL/Kg of body weight) and blood samples were taken from the tail vein before fat load (t = 0) and 30, 60, 90, 120, 180, and 240 min after fat administration. Changes in serum lipids, glucose, insulin, and glucagon levels were measured at different time-points. Expression of liver and adipose tissue metabolic genes were also determined before (t = 0) and after the fat load (t = 240 min). Postprandial dyslipidemia progressively increased with ageing and this could be associated with hepatic ChREBP activity. Interestingly, moderate chronic FR reduced adiposity and avoided excessive postprandial hypertriglyceridemia in 7- and 24-month-old Wistar rats, strengthening the association between postprandial triglyceride levels and adiposity. The 24-month-old rats needed more insulin to maintain postprandial normoglycemia; nevertheless, hyperglycemia occurred at 240 min after fat administration. FR did not alter the fasted serum glucose levels but it markedly decreased glucagon excursion during the OLLT and the postprandial rise of glycemia in the 24-month-old rats, and FGF21 in the 7-month-old Wistar rats. Hence, our results pointed to an important role of FR in postprandial energy metabolism and insulin resistance in ageing. Lastly, our data support the idea that the vWAT might function as an ectopic site for fat deposition in 7-month-old and in 24-month-old Wistar rats that could increase their browning capacity in response to an acute fat load.

## 1. Introduction

Nowadays, prevention of primary diseases and maintenance of health in the population, through a healthy nutrition, has become of great interest, in order to maintain the sustainability of healthcare systems worldwide [1]. In this sense, aged people have a considerable public health importance due to the cost management in treating older adult patients with multiple co-morbid conditions [2]. Abnormal lipid metabolism or dyslipidemia, non-alcoholic fatty liver disease (NAFLD), obesity, and insulin resistance are common in adult and aged people and increase the risk for other liver disorders, diabetes, and cardiovascular diseases, which remains the major cause of worldwide morbidity and mortality in the elderly population, despite scientific and therapeutic advances [3]. Although, postprandial lipemic behavior varies according to different age stages, the mechanism underlying this effect is still unknown [4].

Until recently, excessive postprandial dyslipidemia after fat load have been attributed to the increased levels of triglyceride-rich lipoprotein (TRL) of intestinal origin [4,5]. Currently, it has been clearly demonstrated that VLDL (very low density lipoproteins) remnants are the major that are TRL increased postprandially, after a fat meal [6,7,8,9]. Nevertheless, the extent and duration of postprandial dyslipidemia in aged organisms have also been associated with changes in the absorptive processes, with reduced activity of lipoprotein lipases (LPL) and a decreased rate of clearance of TRL remnants by receptor-mediated processes [5,10], although the available data are still inconclusive. 

In the setting of NAFLD, postprandial dyslipidemia have been attributed to the state of insulin resistance, which increases the rate of peripheral lipolysis, and the rate of TG synthesis and VLDL secretion by the liver [11,12]. Increased hepatic secretion and impaired clearance of VLDL are associated with high plasma concentrations of TG and low density lipoproteins (LDL), two risk markers of the metabolic syndrome, T2DM, and cardiovascular diseases [13]. Moreover, insulin resistance elevates plasma levels of NEFA. In this condition, the insulin-stimulated glucose uptake in skeletal muscles is reduced and the production of fatty acid derivates, such as long-chain acyl-CoA, DAG and ceramides, which promote oxidative stress, inflammation, and impaired insulin signaling, are increased, resulting in postprandial hyperglycemia [14].

Conversely, a recent report proposed that postprandial dyslipidemia is the main cause of insulin resistance in the obese state [15]. In this sense, excessive VLDL remnants in the postprandial state favors adipocyte hypertrophy and inflammation of visceral adipose tissue (vWAT), the prominent causes of insulin resistance and the syndromes associated with insulin resistance, such as NAFLD [15]. However, a significant gap of knowledge exists between the underlying cellular and molecular mechanisms leading to postprandial dyslipidemia and insulin resistance in aged organisms. 

In the present work, we aim to investigate the progression of postprandial dyslipidemia in a rat model of insulin resistance associated with ageing. The study was implemented in mature middle-aged (7 months) and middle-old-aged (24 months) Wistar rats, which exhibit increased adiposity and an altered lipid profile when fasting together with liver steatosis and fibrosis, but avoid the onset of diabetes mellitus [16,17,18]. Interestingly, in 24-month-old rats, the liver transcription factor ChREBP localizes at a nuclear fraction, even in the fasted state [16]. In addition, it has been reported that ChREBP overexpression in mice is able to integrally induce the lipogenic and esterification program in the liver, increasing the hepatic content of monounsaturated fatty acids, without alterations in insulin sensitivity, despite liver steatosis [19]. Hence, we hypothesized that ChREBP play a pivotal role in the development of fasting and postprandial hypertriglyceridemia in Wistar rats, during ageing. Moreover, because food restriction (FR) improved the systemic insulin sensitivity in 7-month-old but not in 24-month-old Wistar rats [17], we speculate that moderate chronic FR for 3 months would modify hepatic ChREBP expression and avoid postprandial dyslipidemia, at least in 7-month-old mature middle-aged rats. 

Since the most functional assessment of postprandial lipid metabolism involves ingestion of a high-fat meal, we performed an OLLT, using extra-virgin olive oil, in order to evaluate the effect of ageing and chronic FR in the time course of postprandial triglyceridemia, cholesterolemia, glycemia, glucagonemia, and insulinemia, in 7-month-old and 24-month-old Wistar rats. Furthermore, to determine the metabolic impact of the acute fat-overload on ChREBP activity, we determined the expression pattern of ChREBP isoforms in liver and adipose tissues. Additionally, we examined the expression of pivotal genes involved in fatty acid and lipoprotein uptake, lipogenesis, lipolysis, fat oxidation, and thermogenesis in these tissues, as well as the expression of the ChREBP target gene FGF21, because recent reports demonstrated that postprandial dyslipidemia and fatty liver might modulate postprandial FGF21 levels in humans and mice [20,21]. 

The data presented herein indicate that postprandial hypertriglyceridemia, progressively increases with ageing and could be associated with the conservation of hepatic ChREBP activity and fatty acid esterification program. Interestingly, moderate chronic FR avoids excessive postprandial hypertriglyceridemia in both 7-month-old food-restricted (7mFR) and 24-month-old food-restricted (24mFR) Wistar rats, supporting the notion that postprandial TG levels firstly depend on the degree of adiposity. Nevertheless, we speculated that FR could improve dyslipidemia in 7mFR and 24mFR rats, through different mechanisms. Finally, using the OLLT we showed that an acute fat load induced molecular signatures of browning in vWAT from old rats, including PRDM16 and the thermogenic gene UCP-1, particularly in old FR rats. Thus, as has been suggested elsewhere [22], these would favor the use of vWAT for ectopic fat deposition in 24-month-old Wistar rats, avoiding the onset of diabetes mellitus.

## 2. Materials and Methods

### 2.1. Animals

The experiments were performed in male 3-, 7-, and 24-month-old Wistar rats from our in-house colony (Centre of Molecular Biology, Madrid, Spain). The maximal life span of the male Wistar rats is about 32–34 months, while their mean life span is about 24 months [23]. Thus, the 24-month-old rats used in the present study are middle-old-aged animals. In addition, according to the previous studies [24] and our previous observations [16,17,18], the 24-month-old rats are not at high risk of mortality and do not present apparent signs of frailty. Nevertheless, 24-month-old rats showed higher intracellular accumulation of lipofuscin than 3-month-old or 7-month-old Wistar rats (see Supplementary Figure S1 for further details) and showed an increased amount of activated stellate cells in the liver [18], both of which are markers of cellular senescence. Animals were housed in climate-controlled quarters with a 12-h light cycle. All rats in this study (fed ad libitum or food-restricted; 8 to 10 animals per group), were fed with a standard chow diet (2014 Teklad Global 14% Protein Rodent Maintenance Diet) (see Supplementary Table S1 for further details) from Harlan Laboratories and water. Animals were handled according to the European Union laws (2010/63/EU) and following the Spanish regulations (RD 53/2013) for the use of laboratory animals. The experimental protocols were approved by the institutional committee of bioethics. All efforts were made to minimize animal suffering and to reduce the number of animals used.

### 2.2. Food Restriction (FR) Protocol

**Scheme 1:**
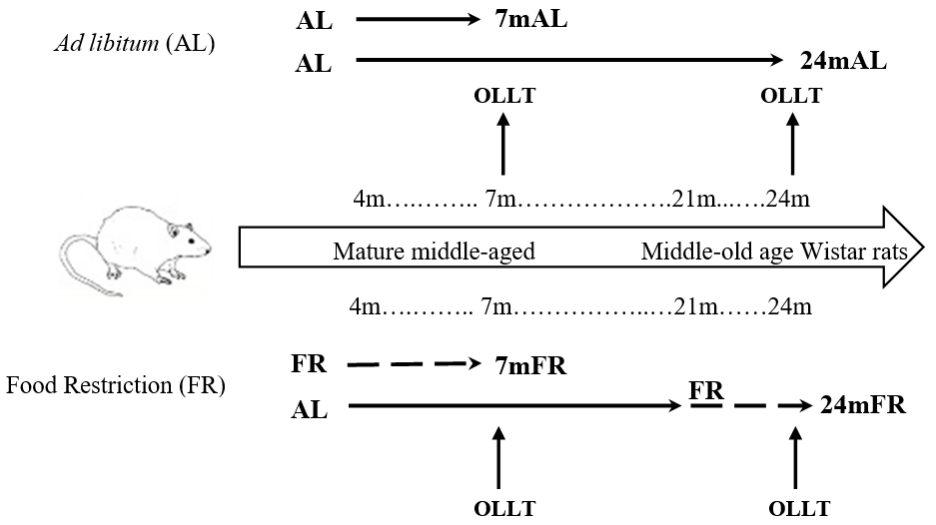
Experimental design.

4- and 21-month-old rats housed individually were fed once daily over 3 months, with an amount of chow equivalent to 75%–80% of their normal food intake, until they reached a reduction of 20% of their body weight, as previously described [17,25]. After 1 month from the start of nutritional restriction, the rats showed a body weight equivalent to ≈80%–85% of ad-libitum-fed aged-mates. After this, they were weighed weekly and the amount of restricted food provided was individually adjusted (Table 1), in order to maintain their body weight for two additional months. Food restricted rats were used at the age of 7 and 24 months (7mFR and 24mFR) (Scheme 1). This FR neither compromised the nutritional status of the animals, nor produced fasting hypoglycemia as we have previously confirmed by OGTT and by the euglycemic-hyperinsulinemic clamp technique, as well as several fasting measurements of glucose levels in the food-restricted animals [17,18].

### 2.3. Oral Lipid Loading Test (OLLT)

The OLLT was performed in 7- and 24-month-old Wistar rats fed ad libitum (AL) or food-restricted (FR) (n = 10 in each of the four experimental groups). Animals were fasted for 16 h (including the overnight period) before the test. A bolus (1 mL/Kg of body weight) of extra-virgin olive oil (aceites Malagón S.L., Ciudad Real, Spain) was orally administered, using a long flexible silicone Abbocath Catheter connected to a sterile polypropylene syringe. Rats remained in their cages without food for the 4 h duration of the study. Blood aliquots from the tail vein at the following times 0, 30, 60, 90, 120, 180, and 240 min were taken. After 240 min, the rats were anesthetized by CO_2_ inhalation, and sacrificed by decapitation. Additionally, 3-month-old AL (3mAL), and 7-month-old and 24-month-old rats fed AL or FR (control groups) were sacrificed before the OLLT after 16 h of fasting. Liver and vWAT were rapidly excised and weighted. The collected tissues were snap-frozen in liquid N_2_ and stored at −80 °C. Serum samples were obtained by blood centrifugation. 

### 2.4. Serum and Tissue Measurements of Hormones and Metabolites

Serum glucose was determined enzymatically, using the AmplexRed Glucose/Glucose Oxidase Assay Kit (Molecular Probes, Inc., Eugene, OR, USA). Serum TG, total-cholesterol, and HDL (high-density lipoprotein) cholesterol levels were measured, respectively, using an enzymatic kit from Stanbio Laboratory (Boerne, TX, USA). The total serum ketones body (KB) and nonesterified fatty acid (NEFA) levels were quantified using an enzymatic kit from Wako Chemicals (Neuss, Germany). Glycerol was quantified by the method of Eggstein and Kuhlmann [26]. Serum levels of FGF21 were determined by a rat-specific ELISA kit from R&D Systems (Minneapolis, MN, USA). Serum insulin and glucagon levels were measured using rat-specific ELISA kits from Mercodia (Uppsala, Sweden) and Wako Chemicals (Neuss, Germany), respectively. Liver and vWAT content of the TG was measured, as previously described [16,25].

### 2.5. Real-Time RT-PCR 

Total RNA was isolated from 100 mg of liver and vWAT using the Trizol reagent (Invitrogen). The cDNA was synthesized from 1.5 mg of DNase-treated RNA. Relative quantitation of peroxisome proliferator-activated receptor alpha (*Ppara*, Rn00566193_m1), peroxisome proliferator-activated receptor gamma coactivator 1-alpha (*Pgc1a*, Rn00580241_m1), aquaporin 7 (*Aqp7*, Rn00569727_m1), fatty acid transporter CD36 (*Cd36*, Rn00580728_m1), lipoprotein lipase (*Lpl*, Rn00561482_m1), low density lipoprotein receptors (*Ldlr*, Rn005998442_m1), LDL receptor related protein 1 (*Lrp1*, Rn01503901_m1), perilipin-1 (*Plin1*, Rn00558672_m1), diacylglycerol O-Acyltransferase 2 (*Dgat2*, Rn01506781_m1), microsomal triglyceride transfer protein (*Mttp*, Rn01522974_m1), carnitine palmitoyl transferase 1a (*Cpt1a*, Rn00676501_g1), carnitine palmitoyl transferase 1b (*Cpt1b*, Rn00566142_m1), fatty acid synthase (*Fas*, Rn01463556_g1), stearoyl CoA desaturase-1 (*Scd1*, Rn00594894_g1), medium-chain acyl-CoA dehydrogenase (*Acadm*, Rn00566390_m1), long-chain acyl-CoA dehydrogenase (*Acadl*, Rn00563121_m1), elongation of very long chain fatty acids 6 (*Elovl6*, Rn00592815_m1), phosphoenolpyruvate carboxykinase 1 (*Pck1*, Rn01529009_g1), glucose-6-phosphatase (*Glu6Pase*, Rn00598561_m1), fibroblast growth factor 21 (*Fgf21*, Rn00590706_m1), glucose transporters 1 (*Glut1*, Rn01417099_m1), 2 (*Glut2*, Rn00563565_m1), and 4 (*Glut4*, Rn00562597_m1), peroxisome proliferator activated receptor gamma (*Pparg*, Rn00440945_m1), tumor necrosis factor alpha (*Tnfα*, Rn99999017_m1), PRD1-BF1-RIZ1 homologous domain-containing 16 (*Prdm16*, Rn01516224_m1), beta-3 adrenergic receptor (*Adrβ3* Rn00565393_m1), and uncoupling protein 1 (*Ucp1*, Rn00562126_m1) mRNA expression analysis from individual mRNA samples of rats of the four experimental groups (n = 8–10 rats per group) were performed on an ABI PRISM 7500 using Pre-Developed TaqMan Assay Reagents (PE Applied Biosystem, Foster City, CA, USA). The relative amount of mRNA in each sample was normalized to that of the reference control 18 S rRNA, using the comparative (2^−ΔCT^) method, according to the manufacturer’s instructions.

Carbohydrate-response element-binding protein isoforms (α and β isoforms) were measured using specific primers from Sigma Aldrich: ChREBP-α: 5′-AGGCTCAAGCATTCGAAGAG-3′ and 5′-TGCATCGATCACAGGTCATT-3′; ChREBP-β: 5′-CTTGTCCCGGCATAGCAAC-3′ and 5′-TCTGCAGATCGCGCGGAG-3′. Quantitative PCR was performed using FAST SYBR GREEN^®^ Master Mix (Applied Biosystems) with normalization of gene expression levels to 18 s.

### 2.6. Calculations and Statistical Analysis 

**Figure 1:**
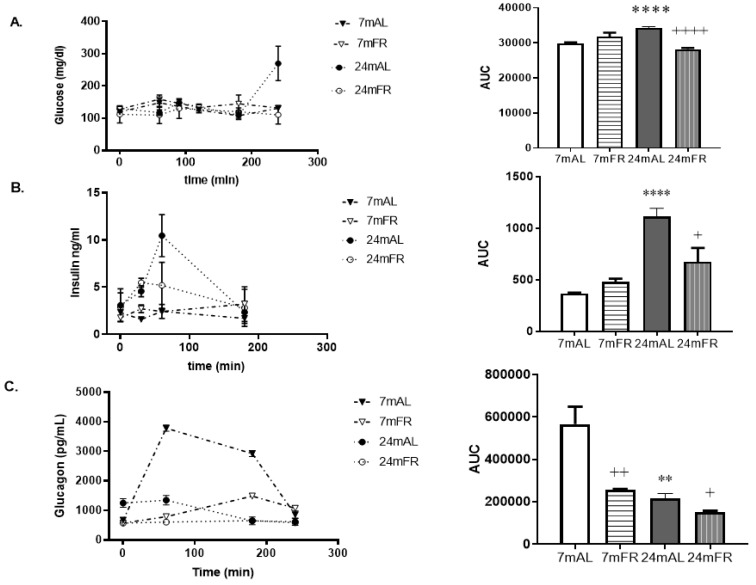
Evolution of serum glucose (**A**), insulin (**B**), and glucagon (**C**) levels in 7- and 24-month-old rats fed ad libitum (AL) or food-restricted (FR) after an oral lipid loading test (OLLT). Blood aliquots from the tail vein were taken at 0, 30, 60, 90, 120, 180, and 240 min after the OLLT. Serum samples obtained by blood centrifugation at each time were pooled and used for the measurement of hormones and metabolites, made in duplicates. Results are the mean ± SEM of 8–10 rats per group. Overall changes in glucose, insulin and glucagon levels during the oral lipid loading test were calculated as the area under the curve (AUC) above the basal level. Unpaired Student’s *t*-test, ** *p* ≤ 0.0025, **** *p* ≤ 0.0001 compared to 7-month-old rats under the same feeding protocol; + *p* ≤ 0.01, ++ *p* ≤ 0.0025, ++++ *p* ≤ 0.0001 compared to the age-matched ad-libitum-fed rats.

**Figure 2:**
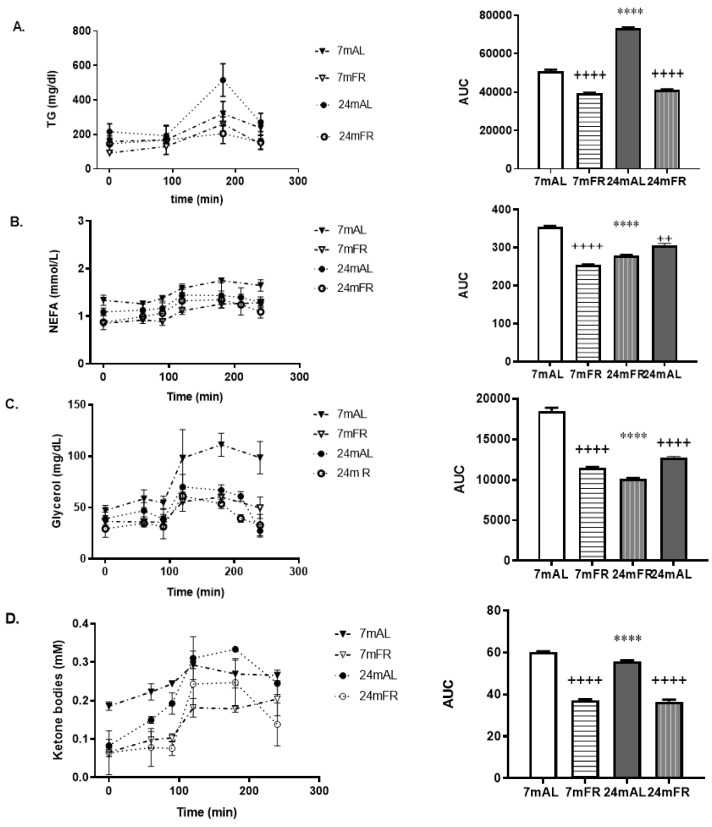
Evolution of serum triacylglycerol (**A**), nonesterified fatty acid (NEFA) (**B**), glycerol (**C**), and ketone body (**D**) levels in 7- and 24-month-old rats fed ad libitum (AL) or food restricted (FR), after an oral lipid loading test (OLLT). Blood aliquots from the tail vein were taken at 0, 30, 60, 90, 120, 180, and 240 min after the OLLT. Serum samples obtained by blood centrifugation at each time were pooled and used for the measurement of hormones and metabolites, conducted in duplicates. Results are the mean ± SEM of 8–10 rats per group. Overall changes in TG, NEFA, Glycerol, and ketone body levels during the oral lipid loading test were calculated as the area under the curve (AUC) above the basal level. Unpaired Student’s *t*-test, **** *p* ≤ 0.0001 compared to 7-month-old rats under the same feeding protocol; ++ *p* ≤ 0.0025, ++++ *p* ≤ 0.0001 compared to age-matched ad libitum fed rats.

**Figure 3:**
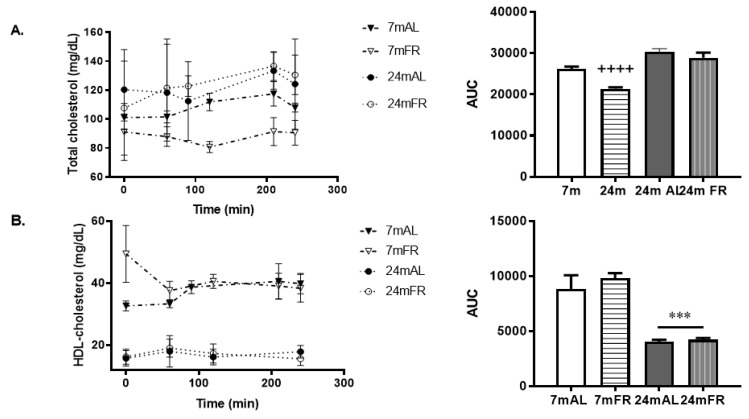
Evolution of serum total cholesterol (**A**) and HDL-cholesterol (**B**) levels in the 7- and 24-month-old rats fed ad libitum (AL) or food restricted (FR), after an oral lipid loading test (OLLT). Blood aliquots from the tail vein were taken at 0, 30, 60, 90, 120, 180, and 240 min after the OLLT. The serum samples obtained by blood centrifugation at each time were pooled and used for the measurement of hormones and metabolites, conducted in duplicates. Results are the mean ± SEM of 8–10 rats per group. Overall changes in the total cholesterol and HDL-cholesterol levels during the oral lipid loading test were calculated as the area under the curve (AUC) above the basal level. Unpaired Student’s *t*-test, *** *p* ≤ 0.001 compared to the 7-month-old rats under the same feeding protocol; ++++ *p* ≤ 0.0001 compared to the age-matched ad-libitum-fed rats.

**Figure 4:**
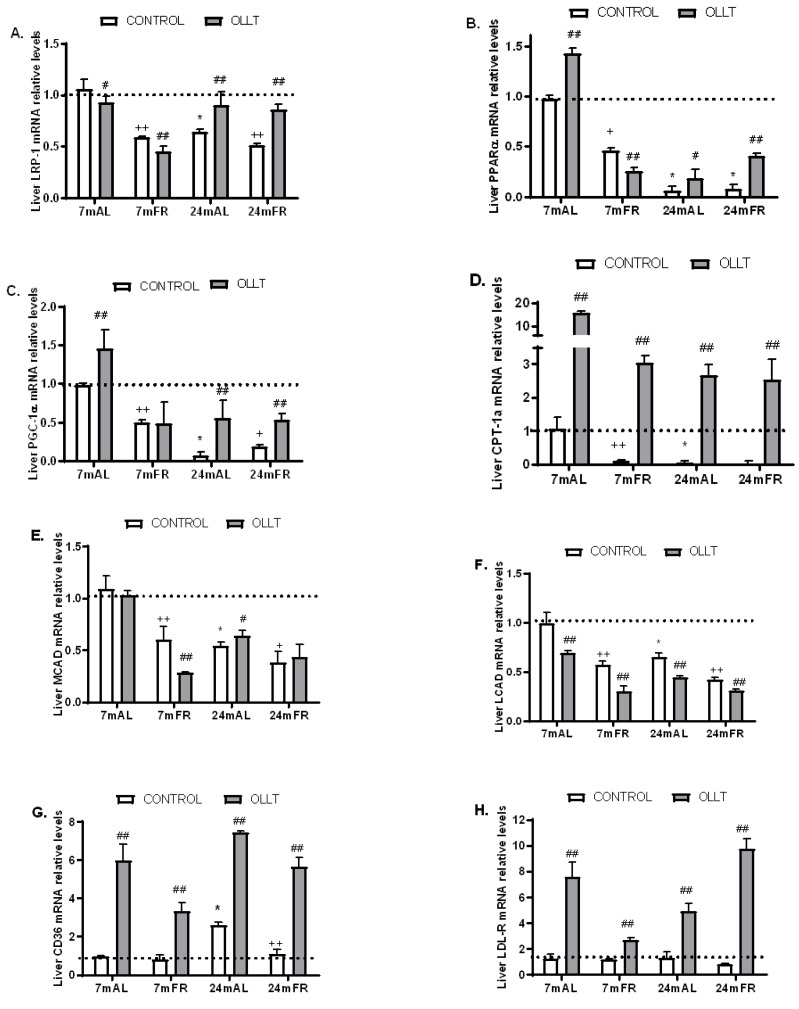
mRNA relative levels of LRP-1 (**A**), PPARα (**B**), PGC-1α (**C**), CPT-1a (**D**), MCAD (**E**), LCAD (**F**), CD36 (**G**), and LDL-R (**H**), in the liver of 7- and 24-month-old rats fed ad libitum (AL) or food-restricted (FR), before (CONTROL) and after an oral lipid loading test (OLLT). Results are the mean ± SEM of 8–10 rats per group, conducted in duplicates. Unpaired Student’s *t*-test, * *p* < 0.05 compared to 7-month-old rats under the same feeding protocol; + *p* < 0.05, ++ *p* < 0.0001 compared to the age-matched ad-libitum-fed rats; # *p* < 0.05, ## *p* < 0.0001 compared to basal levels before a fat load of age-matched rats under the same feeding protocol.

**Figure 5:**
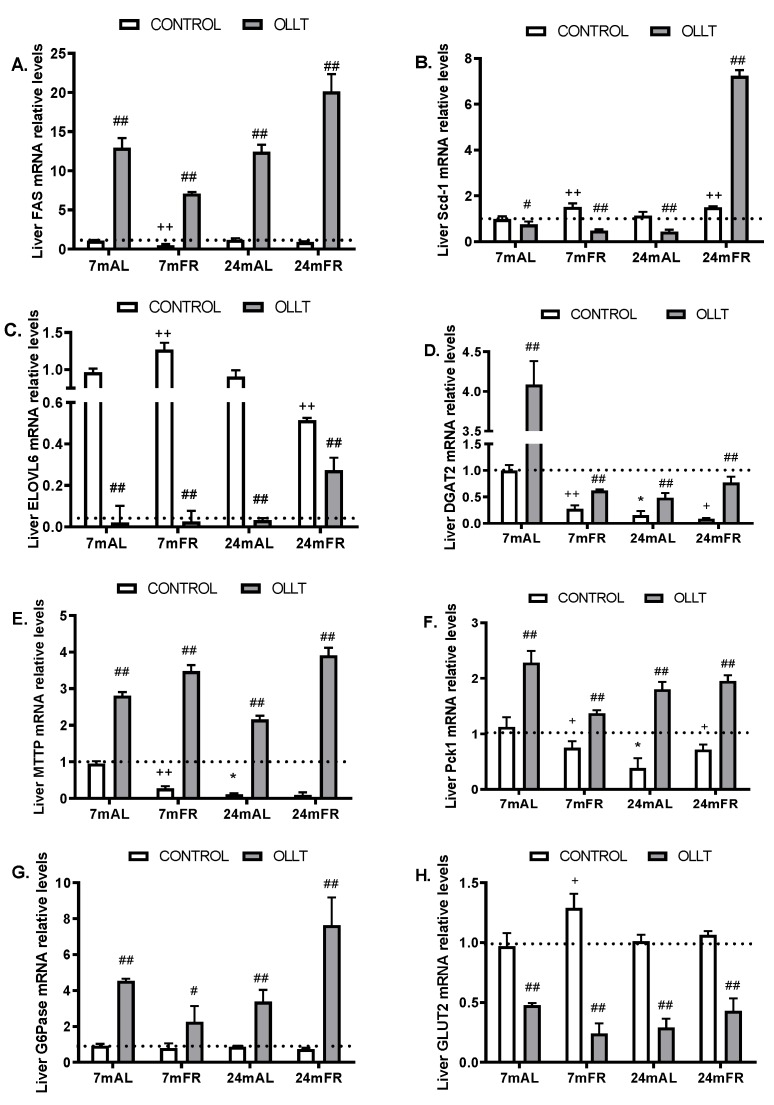
mRNA relative levels of FAS (**A**), Scd-1 (**B**), ELOVL6 (**C**), DGAT2 (**D**), MTTP (**E**), Pck1 (**F**), G6Pase (**G**), GLUT2 (**H**) in the liver in 7 and 24-month old rats fed ad libitum (AL) or food-restricted (FR), before (CONTROL) and after an oral lipid loading test (OLLT). Results are the mean ± SEM of 8–10 rats per group, conducted in duplicates. Unpaired Student’s *t*-test, * *p* < 0.05 compared to the 7-month-old rats under the same feeding protocol; + *p* < 0.05, ++ *p* < 0.0001 compared to the age-matched ad-libitum-fed rats; # *p* < 0.05, ## *p* < 0.0001 compared to the basal levels before a fat load of the age-matched rats under the same feeding protocol.

**Figure 6:**
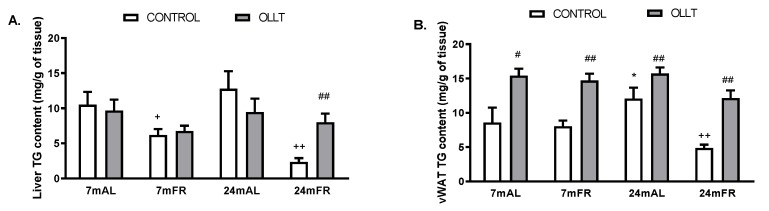
Content of triglycerides (TG) in liver (**A**) and visceral adipose tissue (**B**) in 7- and 24-month-old rats fed ad libitum (AL) or food restricted (FR), before (CONTROL) and after an oral lipid loading test (OLLT). Results are the mean ± SEM of 8–10 rats per group, conducted in duplicates. Unpaired Student’s *t*-test, * *p* < 0.05 compared to the 7-month-old rats under the same feeding protocol; + *p* < 0.05, ++ *p* < 0.0001 compared to the age-matched ad-libitum-fed rats; # *p* < 0.05, ## *p* < 0.0001 compared to the basal levels before a fat load of the age-matched rats under the same feeding protocol.

**Figure 7:**
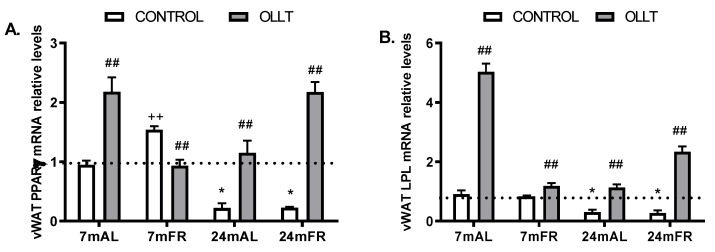

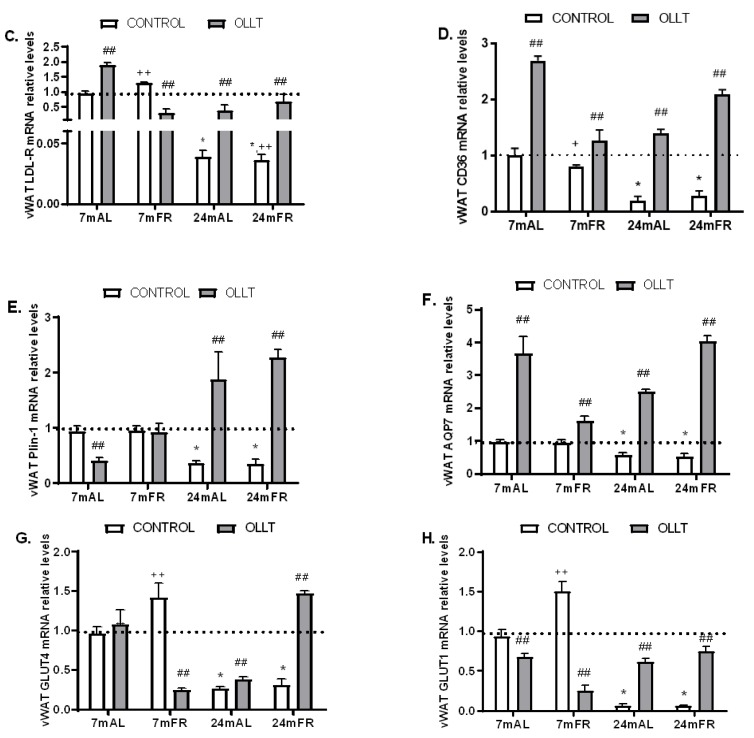
mRNA relative levels of PPARγ (**A**), LPL (**B**), LDL-R (**C**), CD36 (**D**), Plin-1 **E**), AQP7 (**F**), GLUT4 (**G**), and GLUT1 (**H**) in the visceral adipose tissue in the 7- and 24-month-old rats fed ad libitum (AL) or food-restricted (FR), before (CONTROL) and after an oral lipid loading test (OLLT). Results are the mean ± SEM of 8–10 rats per group, conducted in duplicates. Unpaired Student’s *t*-test, * *p* < 0.05 compared to the 7-month-old rats under the same feeding protocol; + *p* < 0.05, ++ *p* < 0.0001 compared to the age-matched ad-libitum-fed rats; ## *p* < 0.0001 compared to the basal levels before a fat load of the age-matched rats under the same feeding protocol.

**Figure 8:**
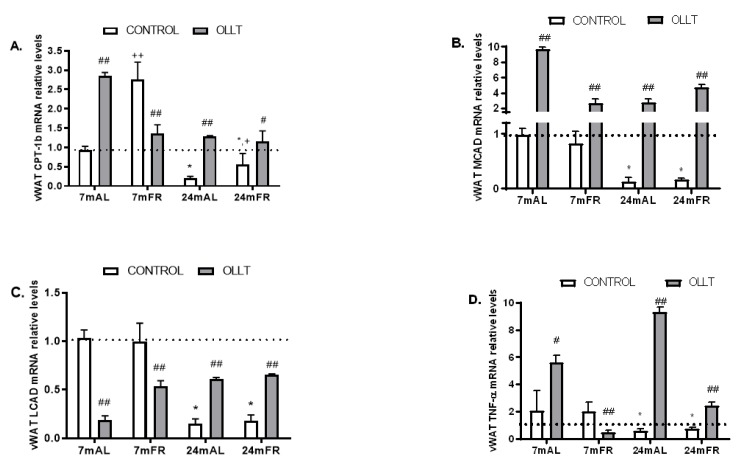

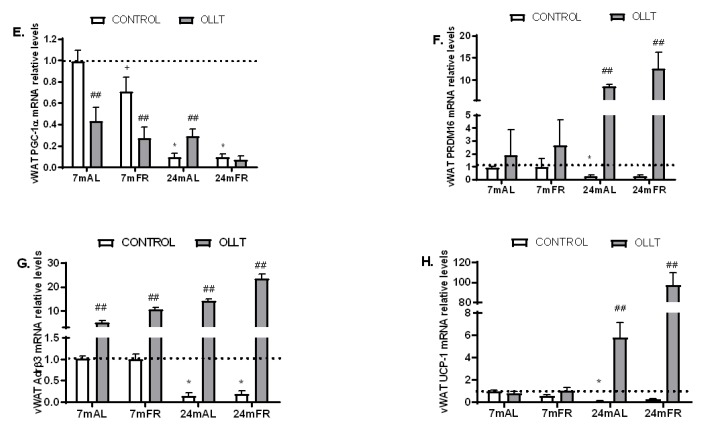
mRNA relative levels of CPT-1b (**A**), MCAD (**B**), LCAD (**C**), TNF-α (**D**), PGC-1α (**E**), PRDM16 (**F**), Adrβ3 (**G**), and UCP-1 (**H**) in the visceral adipose tissue in the 7- and 24-month-old rats fed ad libitum (AL) or food-restricted (FR), before (CONTROL) and after an oral lipid loading test (OLLT). Results are the mean ± SEM of 8–10 rats per group, conducted in duplicates. Unpaired Student’s *t*-test, * *p* < 0.05 compared to the 7-month-old rats under the same feeding protocol; + *p* < 0.05, ++ *p* < 0.0001 compared to the age-matched ad-libitum-fed rats; # *p* < 0.05, ## *p* < 0.0001 compared to the basal levels before a fat load of the age-matched rats under the same feeding protocol.

**Figure 9:**
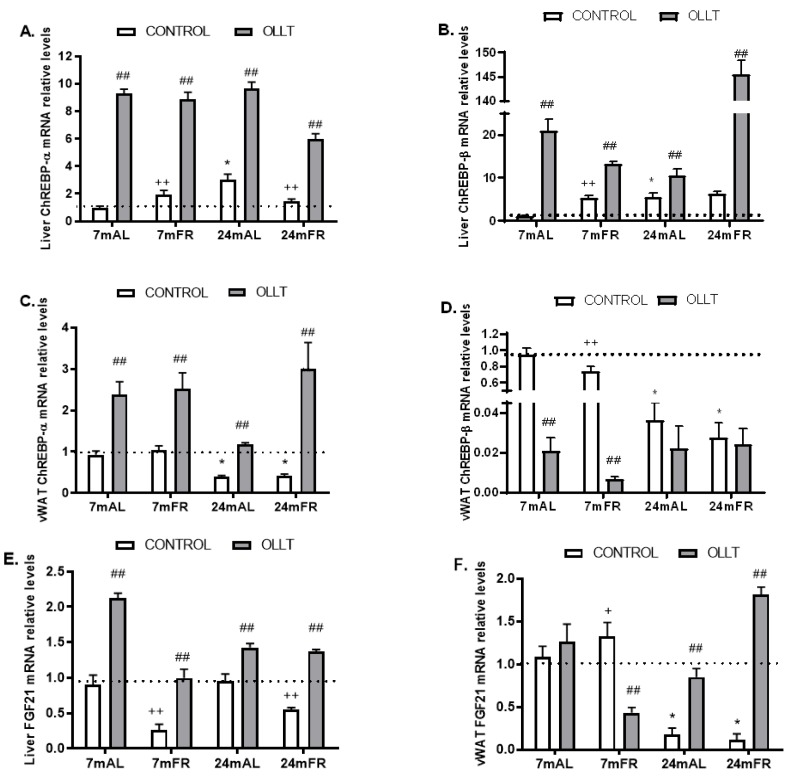
mRNA relative levels of α- and β-ChREBP isoforms in liver (**A**,**B**), and visceral adipose tissue (**C**,**D**), in 7- and 24-month-old rats fed ad libitum (AL) or food restricted (FR), before (CONTROL) and after an oral lipid loading test (OLLT); mRNA relative levels of FGF21 in liver (**E**) and visceral adipose tissue (**F**). Results are the mean ± SEM of 8–10 rats per group, made in duplicate. * *p* < 0.05 compared to 7-month-old rats under the same feeding protocol; + *p* < 0.05, ++ *p* < 0.0001 compared to age-matched ad libitum fed rats; ## *p* < 0.0001 compared to basal levels before a fat load of age-matched rats under the same feeding protocol.

**Figure 10:**
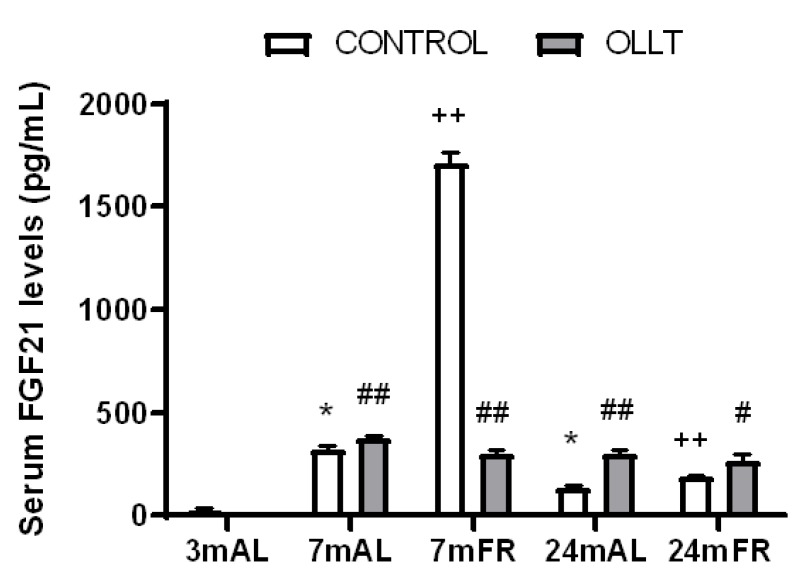
Serum levels of the FGF21 in the 3-, 7-, and 24-month-old rats fed ad libitum (AL) or food-restricted (FR), before (CONTROL) and after an oral lipid loading test (OLLT). Results are the mean ± SEM of 8–10 rats per group, conducted in duplicates. Unpaired Student’s *t*-test, * *p* < 0.05 compared to the 3-month-old rats under the same feeding protocol; ++ *p* < 0.0001 compared to the age-matched ad-libitum-fed rats; # *p* < 0.05, ## *p* < 0.0001 compared to the basal levels before a fat load of the age-matched rats under the same feeding protocol.

Visceral adiposity was the sum of the weight in grams of epidydimal and retroperitoneal fat pads. Insulin Resistance (IR) was evaluated by the homeostasis model assessment method (HOMA-IR), as follows: [fasting insulin (μU/mL) × fasting glucose (mmol/liter)]/22.5 as described [27]. The total response during the OLLT was measured as the areas under the curve (AUCs), which were calculated using the trapezoid rule. Statistical analysis was performed using the GraphPad Prism version 8.2 for Windows (GraphPad Software). Significant differences among groups (3mAL, 7mAL, 7mFR, 24mAL, and 24mFR) were determined by one-way, two-way, or three-way ANOVA, followed by Tukey’s post hoc test. For statistical information about the effects of ageing, diet, OLLT, and their interactions, data have been included in all figures (see Supplementary information to Figure 1, Figure 2, Figure 3, Figure 4, Figure 5, Figure 6, Figure 7, Figure 8, Figure 9 and Figure 10 for further details). For simple comparisons between two groups (7mAL versus 24mAL; 7mAL versus 7mFR; 24mAL versus 24mFR, CONTROL versus OLLT), an unpaired Student’s *t* test was performed. Statistical significance was set at *p* ≤ 0.05.

## 3. Results

### 3.1. Physiological and Metabolic Parameters in the Fasting State 

We have previously shown that 7-month-old and 24-month-old Wistar rats fed AL are characterized by increased adiposity and overall insulin resistance [17]. In addition, we have also reported an increase in leptinemia, triglyceridemia, as well as the presence of fatty liver and hypertrophic adipocytes in these rats (16, 18, 25, and 28). Thus, in agreement with our previous observations, mature middle-aged (7-month-old ad libitum (7mAL)) and middle-old aged (24-month-old ad libitum (24mAL)) Wistar rats exhibit increased body weight, visceral adiposity, and leptinemia when compared to the young Wistar rats (3mAL) (Table 1). Moreover, the HOMA-IR values indicated that the state of insulin resistance was significantly higher in 24mAL than in 7mAL rats (Table 1). 

According to our previous observations [17,25], moderate chronic FR reduced body weight and adiposity in 7-month-old and in 24-month-old rats (Table 1). Nevertheless, moderate chronic FR prevented the increase in HOMA-IR in 7mFR but not in 24mFR old rats (Table 1), confirming the efficiency of the FR at an earlier age [17]. Then, we studied the effect of FR in postprandial lipemic response, during an OLLT performed in rats at two different age stages, 7- and 24-month-old. In order to reduce the impact of the type of meal, a bolus of extra-virgin olive oil (1 mL/Kg of body weight) was given.

### 3.2. Effect of Food Restriction in Serum Glucose, Insulin, and Glucagon Profiles during the OLLT

Before the OLTT (Scheme 1), there were no significant differences observed in the fasting serum levels of glucose and insulin between the four groups of rats used in the OLLT study (Table 2). Nevertheless, basal glucagonemia was markedly increased in the 24mAL rats (Table 2).

During the OLLT, there were no change in the postprandial levels of glucose or insulin in 7mAL and 7mFR old rats (Figure 1A, B). Nevertheless, the maximum glucose response to fat intake was very different in 24mAL rats, because after the fat bolus infusion, these rats exhibited a marked peak of hyperglycemia (Figure 1A). In addition, a peak of hyperinsulinemia was also detected in the 24mAL rats, one hour after fat ingestion (Figure 1B) although the value returned to basal levels at the end of the experiment. Thus, in response to the OLLT, the area under the curves (AUCs) for glucose and insulin were significantly higher in the 24mAL than in the 7mAL rats, and were reduced by FR in the older rats (Figure 1A, B). 

Most interestingly, after fat intake, 7mAL rats showed the maximum postprandial response of glucagon (Figure 1C). This response begins immediately and reached the maximal levels of 60 min after fat ingestion (Figure 1C). Postprandial levels of glucagon were kept higher until 180 min, returning to baseline values at the end of the experiment (Figure 1C). On the other hand, the postprandial levels of glucagon progressively decreased after fat intake in 24-month-old rats fed AL or FR, even though they were lower in 24mFR than in 24mAL rats. Additionally, the AUC data from insulin and glucagon showed inverse responses in these rats (Figure 1B,C). Finally, in the postprandial state, the 7mAL rats achieved both a higher peak in serum glucagon levels as well as a higher overall glucagon concentration than the older rats, in response to the OLLT (Figure 1C).

### 3.3. Serum Lipid Profiles during the OLLT

As expected, serum lipid profile was altered at baseline in the 24-month-old rats compared to the 7-month-old rats (Table 2). FR impaired the increase in TG and NEFA, as well as the glycerol levels in both groups of -7 and 24-month-old rats (Table 2). On the other hand, confirming our previous observations [14], the 24mAL rats showed the lowest values of KB, compared to the 7mAL rats, at the fasting state (Table 2); being similar to those reached in food-restricted rats (Table 2). 

Next, the effects of the OLLT on the serum TG levels are shown in Figure 2A. In the four groups of animals studied, the postprandial peak for TG was observed 180 min after the OLLT, although both the maximum and the total response of TG significantly increased further, at older ages, and decreased with FR (Figure 2A).

The postprandial NEFA, glycerol, and KB responses to the OLLT were similar between groups and started to peak at 90 min, reaching the maximal levels between 100–180 min after fat ingestion (Figure 2B–D). There was a small increase in the NEFA concentration and their levels were close to the respective basal values at 240 min in the four groups of rats studied (Figure 2B). In parallel, KB increased progressively and their levels remained relatively elevated in the four groups of rats, 4 h after the OLLT, compared to their respective basal levels (Figure 2D). Moreover, 4 h after the OLLT, the glycerol concentration returned to their basal levels, only in the 24mAL and 24mFR rats (Figure 2C). In summary, 7mAL and 24mAL showed the maximum rise and the total increase in TG, NEFA, glycerol, and KB levels in the non-fasting state, which are reflected in their respective AUC values (Figure 2B–D). 

On the other hand, total cholesterol levels in the fasted state were higher in the 24mAL rats compared to the 7mAL rats (Table 2), whereas the fasting levels of HDL, the main cholesterol transport lipoprotein in rats [28], decreased in the 24-month-old rats. Moreover, FR induced significant changes in the HDL-cholesterol levels in the 7mFR but not in the 24mFR rats, compared to their AL littermates (Table 2). 

After the OLLT, the postprandial behavior of total cholesterol concentration did not change significantly in the four groups of rats (Figure 3A). However, the total response in cholesterol levels to the fat-rich meal was significantly lower in the 7mFR rats than in the rest of the groups studied (Figure 3A).

In the case of the HDL-cholesterol, we observed a marked drop in the HDL-cholesterol concentration in the 7mFR rats, 60 min after fat ingestion (Figure 3B). Nevertheless, the total response was similar between 7mAL and 7mFR rats and was significantly higher than the total response observed in the 24-month-old rats (Figure 3B).

### 3.4. Changes in the Metabolic Capacity of Liver after the OLLT

Taking into account that delayed lipid absorption processes significantly affects the magnitude of postprandial dyslipidemia in ageing; we decided to study the expression of genes involved in the management of lipids and glucose disposal in liver and vWAT, through quantitative RT-PCR, at baseline and 4 h after the fat load. At baseline, the capacity to remove chylomicrons (CMs) and VLDL-remnants from the blood (LRP-1) was reduced in the 24-month-old rats, when compared to the 7-month-old rats (Figure 4A). In addition, the expression of PPARα, PGC-1α (Figure 4B,C), and those genes involved in beta-oxidation of CPT-1a, MCAD, and LCAD (Figure 4D–F) were lower in the 24-month-old than in the 7-month-old rats. We observed the same trend for the genes involved in fatty acid synthesis and elongation (FAS, Scd-1, ELOVL6) (Figure 5A–C), and those related to glycerol and TG synthesis/VLDL formation and secretion (DGAT2, MTTP, Pck1) (Figure 5D–F), indicating that hepatic lipoprotein and lipid metabolism progressively decays from mature middle-age, in Wistar rats.

On the other hand, after the OLLT, the mRNA levels of genes involved in fatty acid and cholesterol uptake from circulation as CD36 and LDLR were markedly increased in the four groups of animal studied (Figure 4G,H), although the major changes were observed in the 24mFR rats. Additionally, the MCAD mRNA levels were not altered, except in the 7mFR rats, where it was found to be reduced (Figure 4E), whereas the LCAD levels were reduced in all studied rats (Figure 4F). Of note, the reduction in the hepatic oxidative response to a high fat load did not depend on the adiposity. 

Next, we measured the mRNA expression levels of the major genes involved in fatty acid esterification/VLDL formation, in response to the OLLT. As observed in Figure 5A, all groups of rats exhibited enhanced expression of hepatic FAS. Interestingly, the mRNA levels of the Scd-1, DGAT2, and MTTP increased 5-, 10-, and 40-fold, respectively, in the 24mFR rats, compared to their basal levels (Figure 5B,D,E), suggesting an increased capacity for fatty acid esterification in these rats. In line with the gene expression analysis, we found that the hepatic TG levels were markedly increased in the 24mFR rats after the acute fat load (Figure 6A). 

Finally, the mRNA levels of the two genes related to gluconeogenesis, Pck1, and Glu6Pase, were increased (Figure 5F,G), whereas the expression levels of Glut2, which is involved in glucose uptake in the liver, were decreased after the acute lipid load in all groups of rats, compared to their basal levels (Figure 5H). Thus, acute fat ingestion decreased glucose utilization capacity by the liver, while inducing transcription of genes that code for enzymes and factors involved in lipoprotein metabolism, fatty acid esterification, and oxidation, as well as hepatic glucose production.

### 3.5. Changes in the Metabolic Capacity of vWAT after the OLLT

Before the OLLT, the mRNA levels of several genes related to white fat differentiation (PPARγ), hydrolysis of TG in TG-rich lipoproteins, lipoprotein-remnants clearance, and free fatty acids uptake from circulation (LPL, LDLR, and CD36), were significantly lower in the 24mAL and FR rats, when compared to the 7mAL or FR rats (Figure 7A–D). In addition, the expression of genes involved in lipogenic capacity, triglyceride mobilization and glucose uptake (Plin1, AQP7, Glut4, Glut1) (Figure 7E–H), as well as those involved in mitochondrial fatty acid oxidation (CPT-1b, MCAD, and LCAD) (Figure 8A–C), were also decreased in 24mAL and FR rats when compared to the 7mAL or FR rats. These data confirmed that 24 m old rats have lower expression of metabolic genes in vWAT than 7 m old rats, sustaining that basal expression of genes involved in lipid metabolism deteriorates progressively during aging in vWAT independently of obesity. 

Interestingly, fat meal increased the mRNA levels of PPARγ, LPL, LDLR, CD36, Plin1, AQP7, Glut4, and Glut1 (Figure 7A–H) by more than 4-fold and those of CPT-1b, MCAD, and LCAD, in the 24-month-old rats fed AL or FR (Figure 8A–C). Additionally, the mRNA levels of the inflammation marker TNFα in the vWAT were also increased 4 h after the OLLT in the 7mAL, 24mAL, and 24mFR rats (Figure 8D), paralleled to the visceral adiposity index reported in Table 1. 

To investigate whether the lipid overload induced brown fat selective genes in the vWAT, we analyzed gene expression of the PDRM16 and UCP1. Our results showed that PDRM16 and UCP1 mRNA levels increased significantly in the vWAT from 24-month-olds but not in the 7-month-old rats (Figure 8F,H). These results suggest that an oral fat load markedly induced the expression of genes involved in the browning program in 24-month-olds but not in 7-month-old rats, although their metabolic consequences need to be elucidated. Interestingly, in response to fat load, the expression of PPARγ (Figure 7A), LDLR, Glut4, Glut1 (Figure 7C,G,H), as well as CPT-1b, LCAD, TNFα, PGC-1α, PDRM16, and UCP1 (Figure 8A,C–F,H) was not induced in the vWAT from the chronic 7mFR-old rats, suggesting a decreased thermogenic capacity, following the FR in the 7-month-old rats. Nevertheless, the TG storage capacity was significantly increased in these rats (Figure 6B), without any sign of inflammation (Figure 8D). On the other hand, the TG storage capacity was also significantly increased in the 7mAL, 24mAL, and 24mFR rats (Figure 6B). 

### 3.6. Changes in Hepatic and vWAT ChREBP Activity after the OLLT

ChREBP, in liver, promotes lipogenesis and fatty acid esterification [19,29], whereas in adipocytes, regulates the expression of PPARγ target genes involved in thermogenesis and promotes insulin sensitivity [29]. It is known that ChREBPα is translocated from the cytosol to the nucleus upon glucose stimulation, to induce ChREBPβ transcription, which remains localized in the nucleus and has much more potent trans-activity than ChREBPα [29].

In this work, and in agreement with our previous observations reporting that the liver of middle-old age Wistar rats exhibit higher levels of total ChREBP in the nuclear fraction in both fasted and fed state, independently of the levels of the starvation signal ketone bodies [16], we show that the hepatic mRNA levels of both ChREBP isoforms are higher in 24 m than in 7 m old Wistar rats (Figure 9A,B). Interestingly, both ChREBP isoforms were significantly upregulated after 4 h of fat intake in the four investigated groups (Figure 9A,B), indicating that in the liver, ChREBP activity remain higher in the postprandial state despite the lack of carbohydrates in the meal. Of note, ChREBP-β expression increased 25-fold in 24mFR rats (Figure 9B). Then, we suspect that the increases in hepatic triglyceride content in 24mFR rats after the OLLT were due to increased ChREBP-β-mediated hepatic TG synthesis/esterification in these rats (Figure 6A). In contrast to the liver, at baseline, the mRNA levels of both ChREBP isoforms were decreased in vWAT with ageing in Wistar rats (Figure 9C,D). In response to fat load, ChREBP-α mRNA levels increased whereas those of ChREBP-β decreased in vWAT (Figure 9C,D). 

Next, we determined the expression levels of FGF21, a direct target gene of ChREBP, in liver and vWAT, at baseline and after the OLLT. At baseline, hepatic FGF21 mRNA levels were significantly reduced in 7mFR and 24mFR rats (Figure 9E), while in vWAT the mRNA levels of FGF21 changed with age and adiposity (Figure 9F). Fat ingestion increased hepatic mRNA levels of FGF21 in the four groups of rats independently of age and nutritional status (Figure 9E). In vWAT, the intake of fat increased the mRNA levels of FGF21 in 7mAL, 24mAL and 24mFR rats (Figure 9F), rats characterized with the highest values of visceral adiposity (Table 1). This latter finding parallel the increase observed in LDLR, Glut4 and Glut1 mRNA levels in these animals (Figure 7C,G,H). Conversely, the effects of fat ingestion on the expression of FGF21 (Figure 9F) and the genes mentioned above (Figure 7C,G,H) were blunted in vWAT from 7mFR rats, the rats with the lowest values of visceral adiposity (Table 1). 

### 3.7. Changes in the Basal and Postprandial Serum Levels of FGF21 after the OLLT 

The data shown in Figure 10 indicate that in Wistar rats the circulating levels of FGF21 decrease after mature middle-age. Interestingly, baseline serum FGF21 levels in 7mFR rats, with the lowest values of adiposity (Table 1), were particularly high when compared to 7mAL, 24mAL and 24mFR rats, but decreased dramatically after acute fat ingestion (Figure 10). Nevertheless, in rats with higher visceral adiposity index (7mAL, 24mAL, 24mFR) (Table 1), serum FGF21 levels increased 4 h after fat consumption (Figure 10). In order to understand the effect of age on the circulating levels of FGF21 we measured it in young 3-month old rats fed AL. As shown in Figure 10, serum FGF21 levels were low at 3-month, increase progressively until mature middle age and then fall slightly with aging. 

## 4. Discussion

Elevated concentration of circulating lipids after a fat-rich meal is a feature of lipid profile in adult and older individuals, which becomes relevant in the development of obesity and its associated metabolic disorders like type 2 diabetes and liver and cardiovascular diseases. Our previous observations demonstrated that ageing in Wistar rats is associated with increased visceral adiposity, elevated levels of ChREBP and increased lipid deposition in the liver [16,17,18,30]. In this work, we studied the effects of ageing and moderate FR on the development of dyslipidemia in the postprandial state, as well as the transcriptional response of liver and vWAT to the acute fat intake in mature middle-aged and middle-old-aged Wistar rats.

Previously, an oral glucose tolerance test (OGTT) and the euglycemic-hyperinsulinemic clamp technique revealed that 24-month-old Wistar rats need more insulin than 3- and 7-month-old rats, to maintain the serum glucose concentration at euglycemic levels, reflecting peripheral insulin resistance (impaired glucose uptake by adipose tissues and muscle) without diabetes [17]. Herein, the OLLT confirmed these previous observation and brought out another relevant aspect that concerns α-cells in both 7mAL and 24mAL rats, because impaired suppression of glucagon in response to meal challenge is an indicator of insulin resistance in α-cells, as has been reported in diabetic and prediabetic humans [31,32]. In this sense, derangements of α-cells at early age might precede the development of dyslipidemia with ageing in Wistar rats, as suggested in early and recent studies regarding the role of glucagon on lipid metabolism [33].

Moreover, our results clearly showed that postprandial dyslipidemia, mainly hypertriglyceridemia, progressively increased with ageing in Wistar rats, and as suggested by gene expression analysis, it goes in parallel with the progressive decrease in the hepatic capacity to oxidize fatty acids and the progressive decay of the adipose tissue lipogenic capacity, in a situation of metabolic inflammation.

To the best of our knowledge, we found for the first time that hepatic expression of ChREBP-β isoform increased with age and after an acute fat intake in AL or in chronic FR rats. Additionally, the OLLT revealed a coordinated upregulation of ChREBP-β expression and their target genes CD36, LDLR, Glu6Pase, FAS, Scd-1, DGAT2, MTTP, and FGF21 in the liver. Thus, although glucose is the main inductor of ChREBP expression and activity in the liver [34], unexpectedly and despite the lack of glucose in meals, during a fat load, we found a significant induction of ChREBP expression in the liver, in response to an extra-virgin olive oil load.

Previous studies have shown that liver X receptors (LXR) regulate the hepatic expression and transcriptional activity of ChREBP, SREBP-1c, and some lipogenic genes, such as FAS, Scd1, and ELOVL6 [35,36]. Moreover, it has recently been reported that oleic acid, the most abundant component of the extra-virgin olive oil, promotes LXR-dependent hepatic lipogenesis in young mice, increasing the expression and nuclear levels of SREBP-1c, and to a lesser extent, that of ChREBP [37]. Despite this, in 24-month-old rats characterized by insulin resistance and high hepatic TG content, we have previously shown that the total and nuclear levels of ChREBP were markedly up-regulated, while hepatic expression of SREBP-1c and the nuclear levels of its mature form were not altered compared to 3-month-old rats [16]. Thus, according to the data reported herein we suggest that ChREBP expression in the liver, with ageing, could also be modulated by fats, through LXR. Nevertheless, the elucidation of the underlying mechanisms of ChREBP induced by olive oil, with ageing, requires further investigation.

Alternatively, our data suggest that the absence of glucose, during OLLT, might explain the decrease of hepatic GLUT2 expression, and as reported, in liver-specific GLUT2 knockout mice [38], this would not affect the normal glucose output capacity, since gene expression of Glu6Pase was raised and normoglycemia was maintained in the 7mAL/7mFR rats and in the 24mFR rats, and hyperglycemia occurred in the 24mAL rats. Interestingly, in response to the acute fat load, the hepatic ChREBP-β expression increased in all rats studied and their lipogenic target genes markedly increased in the 24mFR rats. Thus, it was tempting to speculate that after the OLLT, intrahepatic carbohydrate metabolites increased and might have signaled the induction of glucose production in a setting of high-fat–zero-carbohydrate meal. Accordingly, these carbohydrate metabolites or the newly synthesized glucose could activate ChREBP and the lipogenic genes that redirect carbons from glucose to glycolysis and lipogenesis, linking endogenous glucose production with lipogenesis, in line with data obtained from high fructose feeding mice and in liver-specific GLUT2 knockout mice [29,38]. Conversely, in the 24mAL rats, the muscle and adipose tissues are resistant to insulin, as we previously demonstrated [17], thus, liver exported glucose and the glycemia rise after the OLLT. Collectively, our results suggest that ChREBP-β might have a role in gluconeogenesis and fatty acid synthesis/esterification, as in mice fed with fructose or with a high fat diet [29,39], reinforcing its role in the development of hepatic steatosis and hypertriglyceridemia, in ageing. In addition, an acute fat load markedly downregulated the ChREBP-β expression in vWAT, which could account for a decreased insulin sensitivity in these animals [40].

**Figure 11:**
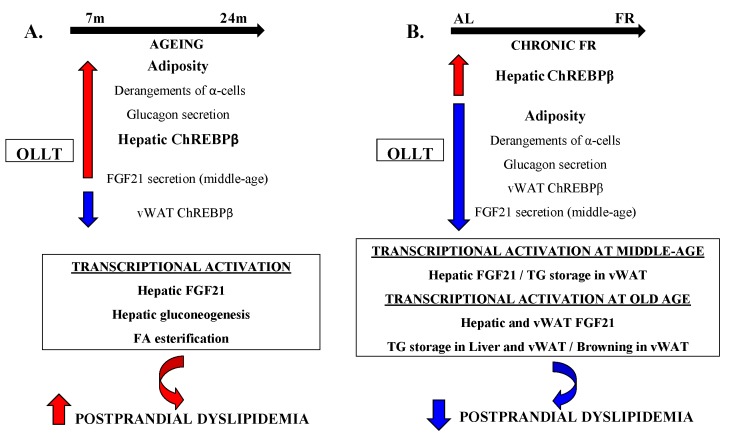
Scheme summarizing the suggested mechanisms about the effects of ageing and moderate chronic food restriction on postprandial dyslipidemia after an oral lipid loading test (OLLT). (**A**) The progression of postprandial dyslipidemia with ageing, in Wistar rats, is associated to increased hepatic ChREBPβ activity and adiposity, as suggested by the gene expression analysis through increased gluconeogenesis and FA esterification in the liver. (**B**). Moderate chronic FR ameliorates postprandial dyslipidemia in middle-aged (7-month-old) and middle-old-aged (24-month-old) Wistar rats, through different mechanisms. In the 7-month-old rats, FR stimulated the transcriptional response leading to an increase in TG accumulation in vWAT, while in the 24-month-old rats, FR stimulated TG accumulation in both liver and in vWAT, and the activation of the browning program in vWAT.

The employment of the OLLT allowed us to infer that moderate chronic food restriction avoids postprandial hypertriglyceridemia in mature middle-aged and also in middle-old-aged Wistar rats, supporting the notion that postprandial TG levels, first of all, depend on the degree of adiposity. Nevertheless, our gene expression data also suggest that FR improves dyslipidemia in 7mFR and 24mFR rats through different mechanisms related to the control of inflammation, insulin responsiveness, and metabolic functionality of adipocytes from vWAT, in mature middle-aged or accumulation of TG in the liver, and induction of vWAT lipogenesis, and thermogenesis in the middle-old aged Wistar rats, respectively (Figure 11). However, the protein modifications associated with the process of ageing [41] unrelated to gene expression should be taken into consideration before a final conclusion might be drawn. 

Additionally, our data showed an increased basal expression of FGF21 in vWAT and its circulating levels in the 7mFR rats, before the OLLT, suggesting that FGF21 in an endocrine/paracrine manner, could coordinate the induction of the lipogenic program of white adipocytes from rats, when adiposity is markedly reduced [42,43]. In addition, FGF21 might influence glucagon secretion in 7mFR rats, since administration of FGF21 decreased the serum levels of glucagon and insulin in ob/ob mice [44]. As expected [45,46], acute fat load upregulates PPARα in the liver of rats with increased adiposity (7mAL, 24mAL and 24mFR rats) and, consequently, the postprandial levels of KB and FGF21 rise in these animals, as a sensor of fat overconsumption.

With regards to FGF21, our study confirmed the findings that FGF21 is induced in obesity [20] through nutritional deprivation [45,46] and through a high-fat diet, in nonhuman primates [47,48]. Fewer data exist that have shown the impact of ageing, per se, on FGF-21, relative to the increased levels with ageing but decreased levels in centenarians [49,50]. In this sense, our results showed that the FGF21 levels in Wistar rats increased progressively in mature middle-age and declined later with aging.

Treatment with FGF21 mimetics, increases thermogenic genes and induces beigeing of white adipose tissue in hamster with increased adiposity, but not in animals with decreased adiposity [51]. Despite the controvert relationship between FGF21 and UCP1 [52], our results suggest that both age and adiposity are implicated. In this sense, it has been demonstrated that FGF21 stimulate lipid disposal and thermogenesis in brown adipose tissue in obesity, while it increases lipoprotein disposal into white adipose tissues in lean mice [41]. Furthermore, although the brown adipose tissue function decreases with ageing [53,54], it is well-known that adipocytes expressing UCP1 are present in white fat of mice and rats, and UCP1 expression is increased after cold exposure or treatment with a beta-adrenoceptor agonist in white adipose tissue, as in typical brown fat [55]. In addition, it has recently been reported that olive oil consumption in mice is associated with upregulation of thermogenesis [56], thus, we propose that the vWAT lipogenic and thermogenic program was markedly induced in old rats, upon an acute high dose of extra-virgin olive oil, suggesting an adaptation against the excess of energy intake, as has been previously reported [57].

Of note, the reduction of postprandial triglyceridemia and glycemia in the 24mFR rats, could also be associated with the dramatic upregulation of PRMD16, FGF21, and UCP1 in vWAT. Then, in contrast to what was observed in subcutaneous adipose tissue in which UCP1 expression and brown-like adipose tissue function were reduced with aging [54], our data suggest the activation of the browning program in the vWAT of older rats. Accordingly, we hypothesized that liver from 24mFR rats could produce a molecular switcher, which determines browning of white adipocytes and overstimulates postprandial thermogenesis to dissipate energy as heat. Similar findings were reported in adenovirus-ChREBP-β-infected mice, although herein we observed that circulating levels of FGF21 did not increase in the 24mFR rats as in adenovirus-ChREBP-β-infected mice [58]. Hence, our findings support the notion that FGF21 acts in an autocrine/paracrine manner in vWAT from aged rats, overstimulating the postprandial thermogenic adaptive responses, as described [59]. 

Finally, based on the analysis about the effects of age, food intake (AL or FR), and their interactions in response to the OLLT, our data support that postprandial lipidemia and expression of genes in liver and vWAT are influenced by age and caloric intake. Additionally, the results of the present work reporting the induction of a thermogenic gene program in vWAT from old Wistar rats could be taken into account among the metabolic adaptations that prevents the aged Wistar rats from becoming overtly obese and diabetic. Expectedly, FR enhanced the increased thermogenic gene response to nutrients observed in the old rats, consistent with its positive effects on this tissue. Probably, the fatty acid composition of extra-virgin olive oil is a major cause of these thermogenic responses but this requires further investigation.

## 5. Conclusions

In summary, our results provide evidence that ageing and overconsumption of fat in aged rats acutely upregulates hepatic ChREBP-β expression and induces transcriptional events in liver and vWAT involved in the regulation of lipid metabolism and postprandial lipidemia. Moreover, moderate chronic FR avoids postprandial hypertriglyceridemia in both mature middle-aged and in middle-old-aged Wistar rats, supporting the notion that postprandial TG levels, first of all, depend on the degree of adiposity. However, the molecular mechanisms that influence the lipid profile in the non-fasting state are quite different between age, as well as the possible intervention of glucagon and FGF21 in the control of dyslipidemia. Although these results have been obtained in a limited low number of tested animals, and not in humans, they could contribute to a better understanding of the effects of caloric restriction and its ability to modify fasting and postprandial dyslipidemia with ageing.

## Figures and Tables

**Table 1:** General characteristics of the ageing model.

Wistar Rats	3mAL	7mAL	7mFR	24mAL	24mFR
Body weight (g)	325 ± 17	425 ± 7 ^&^	382 ± 4 ^+^	646 ±19 ^&,^*	490 ±12 ^&,+^
Mean daily food intake (g)	22 ± 1.2	22 ± 0.4	17 ± 0.5 ^+^	23 ± 0.7	18 ± 0.3 ^+^
Visceral adiposity index (%)	2.1 ± 0.2	5.7 ± 0.3 ^&^	1.2 ± 0.04 ^+^	7.0 ± 0.5 ^&,^*	1.9 ± 0.1 ^+^
Fasting serum leptin (ng/mL)	4.7 ± 0.6	7.5 ± 0.9 ^&^	2.1 ± 0.1 ^+^	16.3 ± 2 ^&,^*	7.1 ± 1.2 ^+^
HOMA-IR	7.5 ± 1.5	7.8 ± 1.9	2.4 ± 0.5 ^+^	10.1 ± 1 ^&,^*	7.3 ± 1.2

Data were obtained from 8–10 rats per group and represent the mean ± SEM of separate determinations. The data were evaluated by one-way ANOVA followed by Tukey’s post hoc test. ^&^ *p <* 0.05 compared to 3-month-old rats; * *p <* 0.05 compared to 7-month-old rats under the same feeding protocol; ^+^ *p <* 0.05 compared to the age-matched ad-libitum-fed rats.

**Table 2:** Baseline levels of hormones and metabolites before the oral lipid loading test (OLLT).

Wistar Rats	7mAL	7mFR	24mAL	24mFR
Fasting serum glucose (mg/dL)	119 ± 5	128 ± 7	131 ± 5	112 ± 22
Fasting serum insulin (ng/mL)	2.0 ± 0.1	1.4 ± 0.9	2.8 ± 0.1	2.1 ± 1
Fasting serum glucagon (pg/mL)	671 ± 29	574 ± 5	1328 ± 62 *	571 ± 8 ^+^
Fasting serum TAG (mg/dl)	156 ± 9	98 ± 14 ^+^	171 ± 8 *	103 ± 4 ^+^
Fasting serum NEFA (mmol/L)	1.27 ± 0.1	0.84 ± 0.002 ^+^	1.08 ± 0.03 *	0.87 ± 0.06 ^+^
Fasting serum glycerol (mg/dl)	47 ± 4	36 ± 0.9 ^+^	39 ± 3 *	24 ± 7 ^+^
Fasting serum KB (mM)	0.18 ± 0.01	0.06 ± 0.01 ^+^	0.081 ± 0.008 *	0.06 ± 0.002 ^+^
Fasting serum Cho (mg/dL)	101 ± 2	91 ± 19	120 ± 13 *	107 ± 16
Fasting serum cHDL(mg/dL)	32.7 ± 1	53.0 ± 14 ^+^	15.8 ± 1 *	16.2 ± 1 *

Data were obtained from 8–10 rats per group and represent mean ± SEM of biochemical determinations made in duplicates. The data were evaluated by one-way ANOVA, followed by Tukey’s post-hoc test. * *p <* 0.05 compared to 7-month-old rats under the same feeding protocol; ^+^ *p <* 0.05 compared to the age-matched ad libitum fed rats.

## Supplementary Materials

The following are available online at https://www.mdpi.com/2072-6643/11/8/1865/s1, Figure S1: Lipofuscin accumulation in hepatic senescent cells with ageing, Table S1: Macronutrients of rodent maintenance diet 2014 Teklad Global 14% protein. Supplementary information to figure 1–10: For statistical information about the effects of ageing, diet, OLLT, and their interactions.

## Abbreviations

OLLT—oral lipid loading test, TG—triglycerides, TRL—triglyceride-rich-lipoprotein, NEFA—non esterified fatty acids, NAFLD—non-alcoholic fatty liver disease, VLDL—very low density lipoprotein, ChREBP—carbohydrate response element binding protein, FGF21—fibroblast growth factor 21, KB—ketone bodies, OGTT—oral glucose tolerance test, DAG—diacylglycerol, vWAT—visceral white adipose tissue, T2DM—type 2 diabetes mellitus, and LXR—liver X receptors.
