# Supplementary material for: Effects of Moderate Chronic Food Restriction on the Development of Postprandial Dyslipidemia with Ageing
Source: Nutrients. 2019 Aug 10;11(8):1865. doi: 10.3390/nu11081865 (PMC6723802; doi:10.3390/nu11081865)
Supplement: Supplementary file 1 [file nutrients-11-01865-s001.pdf]

Effects of moderate chronic food restriction on the development of postprandial dyslipidemia with ageing

Alejandro Fernández[1,2*], Lorena Mazuecos[1,2*], Cristina Pintado[1,3], Blanca Rubio[1,2], Virginia López[1,2], Alain J. de Solís[4], María Rodríguez[1,3], Antonio Andrés[1,2], Nilda Gallardo[1,2#]

SUPPLEMENTARY MATERIALS

Lipofuscin determination

The presence of lipofuscin, a hallmark of senescent cells and particularly of liver oxidative stress, steatosis and fibrosis, was analyzed in unfixed, unstained cryostatic (30 μm thick) liver section from young (3m), mature middle-aged (7m) and middle-old age (24m) Wistar rats. Hepatic lipofuscin and lipofuscin-like pigments autofluorescence was measured at 510-580 nm, with excitation at 488 nm, and was visualized by fluorescent microscopy (Zeiss ICS Standart 25).

young-mature (3m) **middle-aged (7m)** **old (24m)**

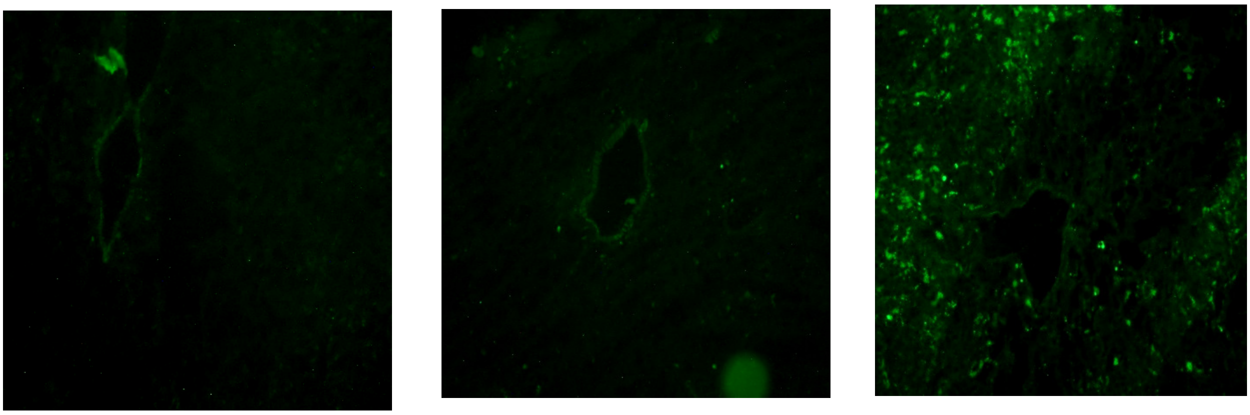

Supplementary Figure S1. Lipofuscin accumulation in hepatic senescent cells with ageing. Four rats from each group were used and 3 images from each rat were analyzed. Representative microphotograps showing autofluorescence detection of the intracellular accumulation of lipofuscin and lipofuscin-like pigments in the liver with ageing.

Supplementary Table S1. Macronutrients of rodent maintenance diet 2014 Teklad Global 14% protein.

Macronutrients	units	quantity
raw protein	%	14.5
lipids	%	4.0
carbohydrates	%	55.5
raw fibers	%	4.5
ash	%	4.7
Metabolizable energy	Kcal/g	3.1
Calories from protein	%	18
Calories from lipids	%	11
Calories from carbohydrates	%	71

Supplementary information to data in Figure 1. Summary of the effects of ageing, diet (AL or FR) and their interactions, obtained by two-way ANOVA followed by Tukey´s post hoc test, on the postprandial levels of glucose, insulin and glucagon in response to OLLT. Statistical significance was set at p≤0.05.

Serum levels	AGE	DIET	AGE & DIET
Glucose AUC	F(1,20)=0.4546<br>**p= 0.5079, ns**	F(1,20)=11.84<br>p= 0.0026	F(1,20)=44.04<br>p≤0.0001
Insulin AUC	F(1,20)=49.15<br>p≤0.0001	F(1,20)=5.96<br>**p= 0.052, ns**	F(1,20)=16.92<br>p= 0.0024
Glucagon AUC	F(1,20)=27.58<br>p≤0.0001	F(1,20)=18.30<br>p= 0.0004	F(1,20)=8.11<br>p= 0.0099

Supplementary information to data in Figure 2. Summary of the effects of ageing, diet (AL or FR) and their interactions, obtained by two-way ANOVA followed by Tukey´s post hoc test, on the postprandial levels of TG, NEFA, glycerol and ketone bodies in response to OLLT. Statistical significance was set at p≤0.05.

Serum levels	AGE	DIET	AGE & DIET
TG AUC	F(1,20)=557.9<br>p≤0.0001	F(1,20)=1802<br>p≤0.0001	F(1,20)=404.6<br>p≤0.0001
NEFA AUC	F(1,20)=10.87<br>p= 0.0036	F(1,20)=299.5<br>p≤0.0001	F(1,20)=103.3<br>p≤0.0001
Glycerol AUC	F(1,20)=184.2<br>p≤0.0001	F(1,20)=337.4<br>p≤0.0001	F(1,20)=69.78<br>p≤0.0001
KB AUC	F(1,20)=18.11<br>p= 0.0004	F(1,20)=1106<br>p≤0.0001	F(1,20)=8.96<br>p= 0.0072

Supplementary information to data in Figure 3. Summary of the effects of ageing, diet AL or FR) and their interactions, obtained by two-way ANOVA followed by Tukey´s post hoc test, on the postprandial levels of TG, NEFA and glycerol in response to OLLT. Statistical significance was set at p≤0.05.

Serum levels	AGE	DIET	AGE & DIET
Total Cholesterol AUC	F(1,20)=4.44<br>p= 0.0479	F(1,20)=56.28<br>p≤0.0001	F(1,20)=16.50<br>p= 0.0006
HDL- Cholesterol AUC	F(1,20)=59.24<br>p≤0.0001	F(1,20)=0.78<br>**p= 0.3853, ns**	F(1,20)=0.34<br>**p= 0.5642, ns**

Supplementary information to data in Figure 4. Summary of the effects of ageing, diet (AL or FR), OLLT and their interactions, obtained by three-way ANOVA followed by Tukey´s post hoc test, on the expression of liver genes involved in lipid uptake and oxidation. Statistical significance was set at p≤0.05.

Gene	OLLT	AGE	DIET	OLLT & AGE	OLLT & DIET	AGE & DIET	OLLT, AGE & DIET
LRP-1	F(1,50)=19.36 p≤0.0001	F(1,50)=2.74 **p = 0.104, ns**	F(1,50)=216.9 p≤0.0001	F(1,50)=133.5 p≤0.0001	F(1,50)=1.147 **p = 0.289, ns**	F(1,50)=103.5 p≤0.0001	F(1,50)=2.134 **p = 0.150, ns**
PPARα	F(1,50)=553 p≤0.0001	F(1,50) =2039 p≤0.0001	F(1,50) =85.30 p≤0.0001	F(1,50)=1639 p≤0.0001	F(1,50) =72.24 p≤0.0001	F(1,50) =0.009 p = 0.987	F(1,50) =2658 p≤0.0001
PGC-1α	F(1,48)=74.95 p≤0.0001	F(1,48)=186.0 p≤0.0001	F(1,48)=81.07 p≤0.0001	F(1,48)=5.83 p= 0.0196	F(1,48)=17.02 p≤0.0001	F(1,48)=104.1 p≤0.0001	F(1,48)=5.34 p= 0.0249
CPT-1a	F(1,48)=3876 p≤0.0001	F(1,48)=1610 p≤0.0001	F(1,48)=1428 p≤0.0001	F(1,48)=1161 p≤0.0001	F(1,48)=1046 p≤0.0001	F(1,48)=1365 p≤0.0001	F(1,48)=1003 p≤0.0001
MCAD	F(1,50)=6.92 p= 0.0113	F(1,50)=128.9 p≤0.0001	F(1,50)=321.9 p≤0.0001	F(1,50)=35.80 p≤0.0001	F(1,50)=12.05 p= 0.0001	F(1,50)=96.32 p≤0.0001	F(1,50)=5.72 p=0.0205
LCAD	F(1,50)=323.8 p=0.0113	F(1,50)=222 p≤0.0001	F(1,50)=583.7 p≤0.0001	F(1,50)=28.07 p≤0.0001	F(1,50)=6.43 p= 0.0144	F(1,50)=79.80 p≤0.0001	F(1,50)=1.54 **p=0.2191, ns**
CD36	F(1,48)=1668 p≤0.0001	F(1,48)=189.1 p≤0.0001	F(1,48)=214.5 p≤0.0001	F(1,48)=20.82 p≤0.0001	F(1,48)=45.70 p≤0.0001	F(1,48)=1.20 **p= 0.2786, ns**	F(1,48)=28.90 p≤0.0001
LDL-R	F(1,50)=1204 p≤0.0001	F(1,50)=49.27 p≤0.0001	F(1,50)=1.07 **p= 0.3059, ns**	F(1,50)=63.49 p≤0.0001	F(1,50)=0.485 **p= 0.4891, ns**	F(1,50)=247.8 p≤0.0001	F(1,50)=297.3 p≤0.0001

Supplementary information to data in Figure 5. Summary of the effects of ageing, diet (AL or FR), OLLT and their interactions, obtained by three-way ANOVA followed by Tukey´s post hoc test, on the expression of liver genes involved in lipogenesis, fatty acid oxidation and glucose disposal. Statistical significance was set at p≤0.05.

Gene	OLLT	AGE	DIET	OLLT & AGE	OLLT & DIET	AGE & DIET	OLLT, AGE & DIET
FAS	F(1,50)=2034 p≤0.0001	F(1,50)=145.1 p≤0.0001	F(1,50)=0.839 **p= 0.3639, ns**	F(1,50)=122.0 p≤0.0001	F(1,50)=6.48 p= 0.0140	F(1,50)=162.6 p≤0.0001	F(1,50)=147.8 p≤0.0001
Scd-1	F(1,50)=626.9 p≤0.0001	F(1,50)=1903 p≤0.0001	F(1,50)=2427 p≤0.0001	F(1,50)=1751 p≤0.0001	F(1,50)=1401 p≤0.0001	F(1,50)=2096 p≤0.0001	F(1,50)=2297 p≤0.0001
ELOVL6	F(1,48)=2315 p≤0.0001	F(1,48)=65.35 p≤0.0001	F(1,48)=5.54 p=0.0227	F(1,48)=244.7 p≤0.0001	F(1,48)=23.27 p≤0.0001	F(1,48)=45.05 p≤0.0001	F(1,48)=184.9 p≤0.0001
DGAT2	F(1,48)=1161 p≤0.0001	F(1,48)=1173 p≤0.0001	F(1,48)=919 p≤0.0001	F(1,48)=340.4 p≤0.0001	F(1,48)=333.3 p≤0.0001	F(1,48)=1133 p≤0.0001	F(1,48)=559.7 p≤0.0001
MTTP	F(1,48)=8885 p≤0.0001	F(1,48)=114.1 p≤0.0001	F(1,48)=221.7 p≤0.0001	F(1,48)=48.06 p≤0.0001	F(1,48)=729.0 p≤0.0001	F(1,48)=226.8 p≤0.0001	F(1,48)=13.48 p≤0.0001
Pck1	F(1,50)=946.8 p≤0.0001	F(1,50)= 21.52 p≤0.0001	F(1,50)=30.79 p≤0.0001	F(1,50)=36.75 p≤0.0001	F(1,50)=24.76 p≤0.0001	F(1,50)=150.6 p≤0.0001	F(1,50)=6.02 p=0.0177
G6Pase	F(1,50)=319.9 p≤0.0001	F(1,50)=25.54 p≤0.0001	F(1,50)=4.20 p= 0.0456	F(1,50)=28.35 p≤0.0001	F(1,50)=7.66 p= 0.0079	F(1,50)=65.14 p≤0.0001	F(1,50)=65.20 p≤0.0001
GLUT2	F(1,50)=1062 p≤0.0001	F(1,50)=4.00 **p= 0.0509, ns**	F(1,50)=9.37 p= 0.0035	F(1,50)=4.38 p= 0.0413	F(1,50)=27.96 p≤0.0001	F(1,50)=1.47 **p= 0.2298, ns**	F(1,50)=51.51 p≤0.0001

Supplementary information to data in Figure 6. Summary of the effects of ageing, diet (AL or FR), OLLT and their interactions, obtained by three-way ANOVA followed by Tukey´s post hoc test, on TG content in liver and vWAT. Statistical significance was set at p≤0.05.

Tissue	OLLT	AGE	DIET	OLLT & AGE	OLLT & DIET	AGE & DIET	OLLT, AGE & DIET
Liver TG	F(1,30)=1.02 **p= 0.3207, ns**	F(1,30)=0.073 **p= 0.7883, ns**	F(1,30)=87.29 p≤0.0001	F(1,30)=1.58 **p= 0.218**	F(1,30)=25.71 p≤0.0001	F(1,30)=5.20 p= 0.0298	F(1,30)=13.68 p= 0.0009
vWAT TG	F(1,30)=244.2 p≤0.0001	F(1,30)=1.49 p= 0.2304	F(1,30)=59.54 p≤0.0001	F(1,30)=2.74 p= 0.1078	F(1,30)=4.84 p= 0.0353	F(1,30)=36.95 p≤0.0001	F(1,30)=5.78 p= 0.0223

Supplementary information to data in Figure 7. Summary of the effects of ageing, diet (AL or FR), OLLT and their interactions, obtained by three-way ANOVA followed by Tukey´s post hoc test, on the expression of vWAT genes involved in lipid disposal and glucose uptake. Statistical significance was set at p≤0.05.

Gene	OLLT	AGE	DIET	OLLT & AGE	OLLT & DIET	AGE & DIET	OLLT, AGE & DIET
PPARγ	F(1,58)=674.8 p≤0.0001	F(1,58)=184.2 p≤0.0001	F(1,58)=7.47 p= 0.0083	F(1,58)=279.2 p≤0.0001	F(1,58)=36.77 p≤0.0001	F(1,58)=156.1 p≤0.0001	F(1,58)=448.6 p≤0.0001
LPL	F(1,58)=3172 p≤0.0001	F(1,58)=875.4 p≤0.0001	F(1,58)=439.0 p≤0.0001	F(1,58)=144.1 p≤0.0001	F(1,58)=371.9 p≤0.0001	F(1,58)=1509 p≤0.0001	F(1,58)=1454 p≤0.0001
LDL-R	F(1,58)=60.56 p≤0.0001	F(1,58)=750.6 p≤0.0001	F(1,58)=64.94 p≤0.0001	F(1,58)=80.25 p≤0.0001	F(1,58)=188.1 p≤0.0001	F(1,58)=164.8 p≤0.0001	F(1,58)=341.0 p≤0.0001
CD36	F(1,58)=2403 p≤0.0001	F(1,58)=297.5 p≤0.0001	F(1,58)=64.27 p≤0.0001	F(1,58)=69.87 p≤0.0001	F(1,58)=34.11 p≤0.0001	F(1,58)=531.7 p≤0.0001	F(1,58)=306.9 p≤0.0001
Plin-1	F(1,58)=184.1 p≤0.0001	F(1,58)=61.25 p≤0.0001	F(1,58)=18.52 p≤0.0001	F(1,58)=356.3 p≤0.0001	F(1,58)=17.73 p≤0.0001	F(1,58)=0.541 **p= 0.4646, ns**	F(1,58)=0.187 **p= 0.6663, ns**
AQP7	F(1,58)=2564 p≤0.0001	F(1,58)=7.38 p= 0.0087	F(1,58)=11.18 p= 0.0015	F(1,58)=141.2 p≤0.0001	F(1,58)=7.34 p= 0.0088	F(1,58)=425.7 p≤0.0001	F(1,58)=446.3 p≤0.0001
GLUT4	F(1,58)=6.09 p= 0.0165	F(1,58)=184.4 p≤0.0001	F(1,58)=64.90 p≤0.0001	F(1,58)=627.6 p≤0.0001	F(1,58)=6.98 p= 0.0106	F(1,58)=262.7 p≤0.0001	F(1,58)=622.5 p≤0.0001
GLUT1	F(1,58)=17.95 p≤0.0001	F(1,58)=874.4 p≤0.0001	F(1,58)=17.30 p≤0.0001	F(1,58)=1876 p≤0.0001	F(1,58)=185.5 p≤0.0001	F(1,58)=0.008 **p= 0.9257, ns**	F(1,58)=314.4 p≤0.0001

Supplementary information to data in Figure 8. Summary of the effects of ageing, diet (AL or FR), OLLT and their interactions, obtained by three-way ANOVA followed by Tukey´s post hoc test, on the expression of vWAT genes involved in lipid oxidation, browning and inflammation. Statistical significance was set at $p \leq 0.05$.

Gene	OLLT	AGE	DIET	OLLT & AGE	OLLT & DIET	AGE & DIET	OLLT, AGE & DIET
CPT-1b	F(1,58)=92.87 p≤0.0001	F(1,58)=433.3 p≤0.0001	F(1,58)=6.33 p= 0.0146	F(1,58)=25.51 p≤0.0001	F(1,58)=281.8 p≤0.0001	F(1,58)=0.247 **p= 0.6206, ns**	F(1,58)=154.4 p≤0.0001
MCAD	F(1,58)=3456 p≤0.0001	F(1,58)=437.4 p≤0.0001	F(1,58)=283.8 p≤0.0001	F(1,58)=118.8 p≤0.0001	F(1,58)=254.6 p≤0.0001	F(1,58)=897.9 p≤0.0001	F(1,58)=824.7 p≤0.0001
LCAD	F(1,58)=21.73 p≤0.0001	F(1,58)=210.1 p≤0.0001	F(1,58)=23.38 p≤0.0001	F(1,58)=775.5 p≤0.0001	F(1,58)=24.04 p≤0.0001	F(1,58)=9.25 p= 0.0350	F(1,58)=21.17 p≤0.0001
TNF-α	F(1,58)=426.8 p≤0.0001	F(1,58)=22.67 p≤0.0001	F(1,58)=387.1 p≤0.0001	F(1,58)=193.8 p≤0.0001	F(1,58)=401.2 p≤0.0001	F(1,58)=6.45 p= 0.0138	F(1,58)=10.09 p= 0.0024
PGC-1α	F(1,58)=93.88 p≤0.0001	F(1,58)=473.6 p≤0.0001	F(1,58)=60.48 p≤0.0001	F(1,58)=188.5 p≤0.0001	F(1,58)=1.15 **p= 0.2867, ns**	F(1,58)=6.09 p= 0.0165	F(1,58)=16.03 p= 0.0002
PRDM16	F(1,58)=225.4 p≤0.0001	F(1,58)=96.89 p≤0.0001	F(1,58)=9.49 p= 0.0032	F(1,58)=134.6 p≤0.0001	F(1,58)=9.10 p= 0.0038	F(1,58)=4.11 p= 0.0047	F(1,58)=4.45 p= 0.0039
Adrβ3	F(1,58)=4920 p≤0.0001	F(1,58)=796.0 p≤0.0001	F(1,58)=409.2 p≤0.0001	F(1,58)=1078 p≤0.0001	F(1,58)=404.8 p≤0.0001	F(1,58)=28.98 p≤0.0001	F(1,58)=27.13 p≤0.0001
UCP-1	F(1,58)=363.8 p≤0.0001	F(1,58)=343.7 p≤0.0001	F(1,58)=287.4 p≤0.0001	F(1,58)=360.8 p≤0.0001	F(1,58)=290.2 p≤0.0001	F(1,58)=289.6 p≤0.0001	F(1,58)=282.4 p≤0.0001

Supplementary information to data in Figure 9. Summary of the effects of ageing, diet (AL or FR), OLLT and their interactions, obtained by three-way ANOVA followed by Tukey´s post hoc test, on the expression of ChREBP isoforms and FGF21 in liver and vWAT. Statistical significance was set at $p \leq 0.05$.

Gene	OLLT	AGE	DIET	OLLT & AGE	OLLT & DIET	AGE & DIET	OLLT, AGE & DIET
Liver ChREBPα	F(1,37)=3662 p≤0.0001	F(1,37)=5.63 p= 0.0229	F(1,37)=118.0 p≤0.0001	F(1,37)=85.14 p≤0.0001	F(1,37)=63.71 p≤0.0001	F(1,37)=174.3 p≤0.0001	F(1,37)=2.89 **p= 0.0971, ns**
Liver ChREBPβ	F(1,45)=14072 p≤0.0001	F(1,45)=7706 p≤0.0001	F(1,45)=8369 p≤0.0001	F(1,45)=6453 p≤0.0001	F(1,45)=7124 p≤0.0001	F(1,45)=9231 p≤0.0001	F(1,45)=10159 p≤0.0001
vWAT ChREBPα	F(1,45)=488.2 p≤0.0001	F(1,45)=42.44 p≤0.0001	F(1,45)=54.64 p≤0.0001	F(1,45)=2.22 **p= 0.1430, ns**	F(1,45)=42.13 p≤0.0001	F(1,45)=32.00 p≤0.0001	F(1,45)=38.97 p≤0.0001
vWAT ChREBPβ	F(1,45)=1568 p≤0.0001	F(1,45)=1436 p≤0.0001	F(1,45)=27.97 p≤0.0001	F(1,45)=1503 p≤0.0001	F(1,45)=22.16 p≤0.0001	F(1,45)=24.84 p≤0.0001	F(1,45)=17.77 p≤0.0001
Liver FGF21	F(1,50) =1208 p≤0.0001	F(1,50) =0.001 **p= 0.9732, ns**	F(1,50) =566.0 p≤0.0001	F(1,50) =50.96 p≤0.0001	F(1,50) =2.42 **p= 0.1255, ns**	F(1,50) =200.9 p≤0.0001	F(1,50) =77.89 p≤0.0001
vWAT FGF21	F(1,58)=215.0 p≤0.0001	F(1,58)=102.0 p≤0.0001	F(1,58)=7.40 **p= 0.0086, ns**	F(1,58)=744.4 p≤0.0001	F(1,58)=0.08 **p= 0.7780, ns**	F(1,58)=174.8 p≤0.0001	F(1,58)=345.1 p≤0.0001

Supplementary information to data in Figure 10. Summary of the effects of ageing, diet (AL or FR), OLLT and their interactions, obtained by three-way ANOVA followed by Tukey´s post hoc test, on serum FGF21 levels. Statistical significance was set at $p \leq 0.05$.

Serum levels	OLLT	AGE	DIET	OLLT & AGE	OLLT & DIET	AGE & DIET	OLLT, AGE & DIET
FGF21	$F(1,26)=774.1$ $p \leq 0.0001$	$F(1,26)=2059$ $p \leq 0.0001$	$F(1,26)=1120$ $p \leq 0.0001$	$F(1,26)=1598$ $p \leq 0.0001$	$F(1,26)=1511$ $p \leq 0.0001$	$F(1,26)=1045$ $p \leq 0.0001$	$F(1,26)=1138$ $p \leq 0.0001$
